# Navigating grief and healthcare: Mixed-methods study on patient experiences of miscarriage during COVID-19

**DOI:** 10.1371/journal.pone.0359158

**Published:** 2026-09-24

**Authors:** Madeline Fernandez-Pineda, Alison Swift, Christyn Dolbier, Melvin Swanson, Marianne Rose Choufani, Kaitlin Guard Banasiewicz

**Affiliations:** 1 Department of Nursing Science, College of Nursing, East Carolina University, Greenville, North Carolina, United States of America; 2 Department of Advanced Nursing Practice and Education, College of Nursing, East Carolina University, Greenville, North Carolina, United States of America; 3 Department of Psychology, Thomas Harriot College of Arts and Sciences, East Carolina University, Greenville, North Carolina, United States of America; 4 College of Nursing, East Carolina University, Greenville, North Carolina, United States of America; Touro University California College of Pharmacy, UNITED STATES OF AMERICA

## Abstract

This paper aims to understand the patient experiences of women who suffered a miscarriage during the COVID-19 pandemic and examine the relationships of patient experience with miscarriage management, psychological distress, and COVID-19-related stressors. A convergent parallel mixed-methods design combined cross-sectional survey data and qualitative descriptive interviews. Seventy-one women in North Carolina who experienced a miscarriage during the COVID-19 stay-at-home mandates were assessed on patient experience, miscarriage management, perceived stress, anxiety, depression, posttraumatic stress, and COVID-19–related stressors. In-depth interviews were conducted with a subsample of 18 participants. Quantitative and qualitative findings of the subsample were integrated using a joint display to examine the level of convergence. Approximately half of participants reported poor patient experiences (52%), and half reported good patient experiences (48%). COVID-19–related stressors, including inability to have a support person present and access to care disruptions, did not differ significantly between groups. Participants reporting poor patient experiences had significantly higher levels of perceived stress, anxiety, and depressive symptoms (p < .05). Among those receiving medical and/or surgical management, good patient experiences were associated with lower psychological distress; this pattern was not observed among participants undergoing expectant management. Qualitative findings identified four categories shaping miscarriage care: Access to Care, Healthcare Provider Interactions, Healthcare Support Staff Interactions, and Healthcare System Interactions. Data integration demonstrated strong convergence for all four subcategories of the Healthcare Provider Interactions category; moderate convergence for access to care; and weak convergence for healthcare support staff interactions and healthcare system interactions. Findings underscore the central role of provider and nursing interpersonal care in shaping patient experiences and psychological well-being following miscarriage, even amid COVID-19-related system disruptions. Results highlight the need for structured follow-up, emotionally supportive care, and attention to organizational policies to improve miscarriage care during public health crises.

## Introduction

Miscarriage, the spontaneous loss of pregnancy before 20 weeks of gestation, affects about 10–20% of pregnancies [1]. This distressing event can cause significant physical and emotional distress, including intense pain, heavy bleeding, hospitalization, and prolonged uncertainty [2]. The emotional impact also includes increased anxiety, depression, post-traumatic stress disorder (PTSD), and feelings of isolation, often exacerbated by inadequate social support and lack of recognition of the loss [3,4].

Women have recognized empathy, sympathy, and reassurance from nurses as crucial to their well-being and long-term coping after miscarriage [5]. Emotional support, referral to support groups/counseling, testing, and suggestions for memorial services (e.g., lighting candles, making necklaces, and naming ceremonies) from healthcare providers have helped provide closure [6]. Despite recognizing the importance of such support, many women still report dissatisfaction with the bereavement care they receive.

*Patient experience* refers to patients’ interactions within the healthcare system, including encounters with doctors, nurses, and other staff, and how well the care provided respects and responds to individual preferences, needs, and values [7]. Women have expressed feeling abandoned and desiring more information, follow-up, sensitivity, emotional support, transparency, and interaction from their providers during and after miscarriage [6,8]. Many felt their experience was dismissed as a routine pregnancy outcome [6]. Women’s psychological distress is often unrecognized by healthcare providers, family, and friends.

One important, yet understudied, component of the miscarriage experience is the management approach offered to patients. While several randomized controlled trials have found no significant differences in psychosocial outcomes or satisfaction between the management type: expectant management (at home waiting for spontaneous expulsion of contents of conception) vs medical management (oral medications to induce expulsion of contents of conception) vs surgical miscarriage management (dilation and curettage) [9,10], the quality of provider interaction during decision-making appears to significantly influence women’s emotional outcomes. Shared decision-making fosters empowerment and individualized care [11,12], particularly when providers demonstrate empathy and support [11]. In contrast, impersonal or dismissive interactions may exacerbate emotional distress [11].

Despite recommendations from the research community, clinicians do not always offer the full range and thorough explanations of management options [13], nor do they consistently provide follow-up care or psychological screening [3,14]. This lack of comprehensive support contributes to the enduring emotional burden following miscarriage. Additionally, research indicates that the quality of care from support staff and the healthcare system impacts patient experience and health outcomes [15–17].

Furthermore, the COVID-19 pandemic introduced additional challenges for women coping with the status of their pregnancy, including disruptions to healthcare practices such as reduced clinic hours and staffing, increased reliance on telehealth, restrictions on support person presence at antenatal visits, and the need to manage or prevent [18,19]. These pandemic-related challenges, particularly limitations on support person presence and delays or changes in access to care, represent distinct stressors that may shape patient experience and psychological distress following miscarriage [20].

Therefore, the aims of this study were to 1) understand the patient experiences of women who miscarried a desired pregnancy during the stay-at-home mandates of the COVID-19 pandemic in North Carolina (March 30, 2020 – February 24, 2021); and 2) examine the relationships of patient experience with miscarriage management, psychological distress (anxiety, perceived stress, depression, and PTSD), and COVID-19-related stressors. We seek to explore women’s perceptions of the psychosocial care they received, identify the specific challenges faced, and provide insights into how healthcare teams and systems can better support them, informing future healthcare practices and policies.

## Methods

### Design

This paper is part of a larger study exploring miscarriage experiences during the COVID-19 pandemic among women in NC. Given the complexity of the topic, different aspects of the study were addressed in separate manuscripts [4,21] to provide a comprehensive analysis. This cross-sectional mixed-methods study employed a convergent parallel design, in which quantitative and qualitative data were collected and analyzed concurrently but separately, then integrated to provide a comprehensive understanding of participants’ experiences [22]. The quantitative component utilized a cross-sectional approach [23], while the qualitative component followed a qualitative descriptive approach [24].

The study was approved by the University and Medical Center Institutional Review Board at East Carolina University, and all participants provided electronic written informed consent for study participation. The research team included nurse scientists with expertise in women’s health and pregnancy loss, one who is bilingual in English and Spanish, a psychologist specializing in perinatal mental health, and a biostatistician.

### Participants and recruitment

Eligible participants were women aged 18 or older who identified as female, experienced a miscarriage (<20 weeks of gestation) of a desired pregnancy between March 30, 2020, and February 24, 2021; spoke and read English or Spanish; and resided in NC. The inclusion of desired pregnancy loss ensured conceptual alignment with the study’s focus on bereavement experiences and psychological distress, as pregnancy intention may shape emotional attachment, coping, and meaning-making following miscarriage. Additionally, Spanish-language study materials were included to reduce language-related barriers and allow participation by eligible Spanish-speaking women, who are often underrepresented in health research.

The research team recruited from May 1 to September 30, 2022, using convenience and snowball sampling. We distributed flyers in English and Spanish with the survey link through 25 NC clinics (22 health departments, two obstetrics and gynecology, and one primary care), 437 Facebook pages (support groups, mom groups, health departments, and community groups), a university faculty/staff listserv, and a major hospital newsletter. Then we invited participants who completed the survey and provided their email for in-depth interviews in the order they were received. After 16 interviews, no new salient themes were emerging. Two additional interviews were conducted to enhance racial and ethnic representation among participants.

### Procedures

The second author translated study materials from English to Spanish using electronic translation software, which were then reviewed and verified for accuracy by the bilingual principal investigator (PI). Participants accessed the online survey in their preferred language, provided electronic informed consent, and completed eligibility screening and study questionnaires. Upon completion, participants received contact information for local and national support groups and mental health resources. In-depth interviews were conducted in English or Spanish and in person (following COVID-19 precautions) or via Microsoft Teams audioconferencing based on participant preference. All interviews were recorded and transcribed using Microsoft Teams. As interviewers, we addressed participant questions, explained participation rights, facilitated the discussion, and documented field notes [25,26]. To foster rapport and create a supportive environment, two of the interviewers disclosed their personal experiences with pregnancy loss, which has been recognized as an effective approach in qualitative research involving sensitive topics and vulnerable populations [27]. Interviewers monitored participants for signs of emotional distress throughout the interview. Participants were reminded that they could pause or discontinue the interview at any time. If distress was observed or expressed, interviewers offered breaks as needed. Following completion of the interview, all participants were emailed information on local and national bereavement support organizations and mental health resources. Interviews lasted between 42 and 112 minutes, and participants only received a $50 debit card after completing the interview.

### Assessments

#### Survey.

The survey assessed miscarriage history (i.e., number, date, weeks’ gestation, location, and management type), reproductive characteristics (e.g., reproductive and infertility treatment history), demographic characteristics, and mental health history (yes/no questions about prior mental health diagnosis and trauma not including pregnancy losses). Key variables included patient experiences, COVID-19-related stressors, and psychological distress specific to miscarriage. Additionally, personal significance of miscarriage, coping strategies, and social support were assessed, but are not addressed by the aims of this paper.

Patient experience. A nine-item measure of patient experience was developed using six items from the Consumer Assessment of Healthcare Providers and Systems Clinician and Group survey (CG-CAHPS) [28] and three items by the researchers. Participants rated the care they received from their healthcare provider (e.g., communication, emotional support, follow-up, etc.) during their pandemic miscarriage on a 3-point scale (1 = No, 2 = Yes, somewhat, 3 = Yes, definitely) for items 1–7. Items 8 and 9 required a “yes/no” response, and item 9 was answered only if participants answered “yes” to item 8. Higher ratings indicated better patient experience. Cronbach’s alpha estimates indicated good internal consistency in this study (α = 0.87).

COVID-19 related stressors. The researchers developed an item to assess ways the COVID-19 pandemic caused stress while experiencing miscarriage. Participants selected any of the 11 stressors that applied to them. For this analysis, we focused on two stressors that most closely reflected healthcare experiences: the ability to bring a support person to healthcare visits and adequate access to medical care.

Miscarriage management. Participants were asked, “How was the miscarriage(s) (< 20 weeks gestation) you experienced between March 30, 2020, and February 24, 2021, managed? Select all that apply.” Response options included: Expectant (I waited for my body to miscarry on its own without medicine or surgery), Surgical (Dilation and curettage procedure, or another surgical procedure), and Medical (a pill, or a medication taken by mouth).

Psychological distress. Participants’ psychological distress from the pandemic miscarriage was assessed using four established scales. Participants were instructed to respond to each measure while reflecting on their COVID-19 pandemic miscarriage. Perceived stress was measured with the 4-item Perceived Stress Scale (PSS-4) [29]. Items use a 5-point scale, from 0 (Never) to 4 (Very often), and are summed after reverse-scoring two items. Scores range from 0 to 16, with higher scores indicating greater perceived stress. The PSS-4 was internally consistent in this study, with Cronbach’s alpha estimate of α = 0.78.

Anxiety symptoms were assessed with the 7-item Generalized Anxiety Disorder Scale (GAD-7) [30]. Items use a 4-point scale from 0 (Not at all) to 3 (Nearly every day). Summed scores are interpreted as: minimal (0–4), mild (5–9), moderate (10–14), and severe anxiety (15–21), with scores ≥ 10 indicating clinically significant anxiety symptoms [30]. Cronbach’s alpha estimates for GAD-7 indicated good internal consistency in this study (α = 0.93).

Depression symptoms were assessed with the 8-item Patient Health Questionnaire (PHQ-8) [31]. Items use a 4-point scale from 0 (Not at all) to 3 (Nearly every day). Summed scores are interpreted as: minimal (0–4), mild (5–9), moderate (10–14), moderately severe (15–19), and severe depression (20–24), with scores ≥ 10 indicating clinically significant depressive symptoms [31]. The PHQ-8 was internally consistent in this study (α = 0.93).

PTSD symptoms were assessed with the 5-item Primary Care PTSD screen for DSM-5 (PC-PTSD-5) [32]. Items use Yes or No options. Three or more “yes” responses indicate probable PTSD [32]. In this study, the PC-PTSD-5 demonstrated acceptable-to-marginal internal consistency (α = 0.68).

#### In-depth interviews.

The first three authors developed a 10-question semi-structured interview guide based on existing research regarding patient-provider interactions during miscarriage [5,6,8,33], the psychological impact of miscarriage [2,3,34], and maternal health stressors during COVID-19 [18,19]. Four questions of our interview guide closely aligned with the aims of this paper including describing: 1) how the COVID-19 pandemic affected participants’ miscarriage experience; 2) their experience if they visited an emergency department (ED) or an obstetrics ED at any time during/after the loss; 3) how the news was delivered or how they found out about their loss and how their miscarriage was managed (including: what was discussed, and if follow-up and mental health resources were offered); and 4) if they wished something about their care had been different. The complete interview guide was previously published as supplementary material [4] and is reproduced as S1 File for the current manuscript.

### Data analysis

#### Survey.

Quantitative data were analyzed using SPSS-28. Descriptive statistics described sociodemographic characteristics, frequencies of the participants responding “yes-definitely/yes” to each of the patient experience items, endorsement of COVID-19 stressors, and mean scores on the psychological distress measures. Miscarriage management was recoded into a binary variable reflecting level of clinical intervention: expectant management only versus any medical and/or surgical management. Because participants could select multiple management types (expectant, medical, surgical), responses were categorized using a hierarchical approach. Participants reporting expectant management only were classified in the expectant group, whereas those reporting any medical and/or surgical management were classified in the medical/surgical group, regardless of whether expectant management was also endorsed. This classification reflected the highest level of clinical intervention received.

A two-step cluster procedure identified two subgroups (good and poor patient experience groups) based on responses to the five most frequently endorsed “yes-definitely/yes” patient experience survey items. A two-step cluster procedure was selected to identify naturally occurring groupings in patient experience responses rather than imposing an a priori cutoff. Chi-square tests for independence examined differences between the two groups in frequencies of those who responded “yes-definitely/yes” on survey items to validate the cluster procedure and illustrate group differences for each patient experience survey item. Independent sample *t*-tests compared the psychological distress scores (PSS-4, PHQ-8, GAD-7, PC-PTSD-5) between the two patient experience groups, and Cohen’s *d* effect sizes were calculated. These same analyses were conducted within each miscarriage management group as well. Chi-square tests compared COVID-19 stressor responses and miscarriage management group between the two patient experience groups, and phi effect sizes were calculated.

#### In-depth Interviews.

We used Microsoft Teams to transcribe the interviews in either English or Spanish, and transcripts were reviewed for accuracy by the first two authors and a bilingual research assistant before analysis. The single Spanish transcript was translated into English using electronic translation software and verified by the bilingual PI. NVivo12 was used to analyze and organize findings. The first and second authors conducted a conventional content analysis, an approach used to describe a phenomenon of interest by allowing categories and sub-categories to emerge directly from the data rather than being predetermined by existing literature or frameworks [35]. This method ensured an inductive, data-driven analysis.

Initially, they independently coded three identical transcripts to generate preliminary codes and categories. Through iterative discussions, they refined these codes to establish a coding framework guided by participant narratives. They then coded a new set of two identical transcripts and met again to compare codes and identify new categories. Each author then independently coded half of the remaining transcripts before reconvening for final discussion and refinement. Through this iterative process, overarching categories and subcategories were inductively developed [35]. The third author assisted in finalizing coding decisions when consensus could not be reached. Method triangulation, including field notes analysis, was conducted to ensure the accuracy and credibility of findings [25].

After completing this initial analysis, we stratified transcripts into two groups based on the quantitative two-step cluster procedure: the good patient experience group (*n* = 12) and the poor patient experience group (*n* = 6). The first, second, and fifth authors then conducted a cross-case analysis using a variable-oriented approach [36] to compare patterns between these two groups. Variables of interest included the quantitative patient experience classification (good vs poor), qualitative evaluative direction (positive vs negative experiences), and qualitatively derived categories and sub-categories from the initial content analysis.

Quantitative and qualitative data were integrated at the case level for the interviewed subsample (n = 18) using a joint display approach [36]. For each category and subcategory, qualitative evaluative direction (positive or negative) was compared with quantitative patient experience classification (good or poor) to assess alignment across cases. A case was considered convergent when a participant classified as having a good patient experience provided a positive qualitative account or when a participant classified as having a poor patient experience provided a negative qualitative account. The convergence percentage was calculated by dividing the number of convergent cases by the total number of participants who discussed the category or subcategory. Participants who did not discuss the category or subcategory were classified as “No Report” and excluded from the convergence calculation rather than treated as neutral. Within each quantitative patient experience group, positive and negative percentages were also calculated among participants who discussed the category or subcategory. Predominant alignment was operationalized as convergence in ≥ 50% of cases. The magnitude of convergence was descriptively categorized as weak (50–59%), moderate (60–74%), or strong (≥75%) to provide a structured interpretation. These thresholds served as heuristic guides for describing alignment between data rather than statistical cutoffs or measures of thematic importance. Integrated findings for the interviewed subsample are presented descriptively in the Results section, with broader interpretive integration addressed in the Discussion.

## Results

### Quantitative findings

Table 1 presents sociodemographic characteristics of good (*n* = 34; 47.9%) and poor (*n* = 37; 52.1%) patient experience groups and the total sample (*N* = 71). Most participants in both patient experience groups were White, not Hispanic or Latina, married, had private insurance, had an annual household income of ≥ $51,000, and lived in rural or suburban areas. Participants in the poor patient experience group were more likely to report annual household incomes ≤ $50,000 compared to those in the good experience group, *Χ*² (1) = 5.438, *p* = .020. Employment status also differed significantly, with participants in the poor experience group more likely to report part-time employment or unemployment compared to those in the good experience group, *Χ*²(1) = 4.262, *p* = .039. No other sociodemographic variables significantly differed between groups. No significant differences were observed in reproductive history variables, including number of pregnancies (*M* = 3.56, *SD* = 1.92), number of living children (*M* = 1.41, *SD* = 1.06), total number of losses (*M* = 1.60, *SD* = .85), losses occurring during the lockdown window (*M* = 1.34, *SD* = .63), or gestational age at first loss (*M* = 8.50, *SD* = 3.11). Additionally, no significant differences were observed between groups in pre-miscarriage mental health diagnoses (*n* = 29, 40.8%) or in prior trauma history (*n* = 33, 46.5%).

**Table 1:** Sociodemographic Characteristics of Survey Participants.

Characteristic	Patient Experience		
	Good (*n* = 34) n(%)	Poor (*n* = 37) n(%)	Total sample (*N* = 71) n(%)
Race			
Black or African American	3(8.8)	3(8.1)	6(8.5)
White	28(82.4)	31(83.8)	59(83.1)
Another race	3(8.8)	3(8.1)	6(8.5)
Ethnicity			
Hispanic or Latina	4(11.8)	1(2.7)	5(7.0)
Not Hispanic or Latina	30(88.2)	36(97.3)	66(93.0)
Marital status			
Married/partnered	32(94.1)	32(86.5)	64(90.1)
Divorced/separated/widowed	2(5.9)	5(13.5)	7(9.9)
Highest educational level			
Less bachelor’s degree	13(38.2)	21(56.8)	34(47.9)
Bachelor’s degree or higher	21(61.8)	16(43.2)	37(52.1)
Annual Household Income			
≤ $50,000	6(17.6)	16(43.2)	22(31.0)
≥ $51,000	28(82.4)	21(56.8)	49(69.0)
Employment			
Full time	23(67.6)	16(14.2)	39(54.9)
Part-time/unemployed	11(32.4)	21(56.8)	32(45.1)
Insurance			
Private	26(76.5)	26(70.3)	52(73.2)
Medicaid/uninsured	8(23.5)	11(29.7)	19(26.8)
Residential Setting			
Rural	15(44.1)	15(40.5)	30(42.3)
Urban	3(8.8)	8(21.6)	11(15.5)
Suburban	16(47.1)	14(37.8)	30(42.3)

Age for the total sample ranged from 18–45 (*M* = 32.7, *SD* = 6.25, *Md = 33*). Age for good patient experience group (*M* = 33.8, *SD* = 5.56, *Md* = 33.5, *n* = 34) and poor patient experience group (*M* = 31.6, *SD* = 6.83, *Md* = 31.0, *n* = 37).

Participants were asked to select the ways that the COVID-19 pandemic caused stress in their lives while experiencing miscarriage(s). Most women in both groups (73.2%) reported they could not take their support person to their healthcare visits, and 14.1% lacked access to medical care due to limited hours or clinics closing. There were no significant differences between the good and poor patient experience groups in inability to bring a support person to visits, *Χ*² (1) = 0.348, *p* = .556, or in lack of access to medical care due to limited hours or clinic closures, *Χ*² (1) = 1.492, *p* = .222.

Table 2 compares patient experience items between the groups, showing significant differences in all items except item nine, “*Did someone from this provider’s office follow up to give you those results?*” As expected, based on the cluster procedure and validating the establishment of the two groups, the good patient experience group had higher “yes, definitely/yes” endorsements across all patient experience items. In the total sample, the lowest endorsements were for “provided information on support groups, therapy, or counseling services” (11.3%) and “provider addressed your emotional needs” (14.1%), and the highest endorsements were for “provider ordered a blood test, x-ray, or other test” (62.0%) and “provider showed respect for what you had to say” (47.9%).

**Table 2:** Frequencies and Chi-Square Results for Endorsement of Patient Experience Survey Items.

Patient experience Item	Total *N* (%)	Patient experience		*Χ* ^*2*^	*df*	*p*	*Phi*
		Good *n* (%)	Poor *n* (%)				
1. Explained things easy to understand	31(43.7)	30(88.2)	1(2.7)	52.70	1	<.001	.86
2. Listened carefully	28(39.4)	28(82.4)	0(0.0)	50.31	1	<.001	.84
3. Showed respect	34(47.9)	32(94.1)	2(5.4)	55.88	1	<.001	.89
4. Spent enough time	22(31.0)	22(64.7)	0(0.0)	34.69	1	<.001	.70
5. Addressed emotional needs	10(14.1)	10(29.4)	0(0.0)	12.67	1	<.001	.42
6. Provided information	8(11.3)	8(23.5)	0(0.0)	9.81	1	.002	.37
7. Scheduled follow-up	26(36.6)	22(64.7)	4(10.8)	22.17	1	<.001	.56
8. Ordered other tests	44(62.0)	26(76.5)	18(48.6)	5.82	1	.016	.29
9. If yes, office followed up^a^	37(84.1)	23(88.5)	14(77.8)	0.91	1	.341	.14

Frequencies and percentages reflect combined endorsement of “Yes, definitely” and “Yes” responses. Total group *N* = 71; good patient experience group *n* = 34; poor patient experience group *n* = 37.

^a^For item 9, *n* = 44 total; *n* = 27 for good group and *n* = 17 for poor group.

In the total sample, 47.9% of participants scored ≥ 10 on the GAD-7, indicating moderate to severe anxiety symptoms (*M* = 9.32, *SD* = 5.85, range = 0–21), 38.0% scored ≥ 10 on the PHQ-8, indicating moderate to severe depressive symptoms (*M* = 8.76, *SD* = 6.94, range = 0–24), and 64.8% had PC-PTSD-5 scores of ≥ 3, consistent with probable PTSD (PC-PTSD-5, *M* = 3.07, *SD* = 1.58, range = 0–5). The mean perceived stress score (PSS-4) was 9.00 (*SD* = 3.41; range = 2–16). Table 3 presents the means, standard deviations, and independent *t*-test results comparing psychological distress scores between the two patient experience groups. The poor patient experience group mean GAD-7 and PHQ-8 scores ≥ 10 and a mean PC-PTSD-5 scores ≥ 3, meeting the established screening thresholds for clinically significant anxiety, depressive symptoms, and probable PTSD. In contrast, the mean scores of the good patient experience group were below the clinical cutoffs for these three measures. The poor patient experience group had significantly higher depression, anxiety, and stress scale scores than participants in the good patient experience group, with medium effect sizes. No significant difference was found in PTSD scores between patient experience groups and effect size was small.

**Table 3:** Comparisons of Psychological Distress Between Good and Poor Patient Experience Groups.

Scale	Patient Experience						
	Total (*N* = 71)	Good (*n* = 34)	Poor (*n* = 37)	*t*	*df*	*p*	*d*
	*M(SD)*	*M(SD)*	*M(SD)*				
PSS4^a^	9.00 (3.41)	8.12 (2.48)	9.81 (3.95)	2.14	69	.036	.51
GAD7^b^	9.32 (5.84)	7.41 (5.09)	11.08 (6.01)	2.76	69	.007	.66
PHQ8^c^	8.76 (6.93)	6.71 (5.57)	10.65 (7.58)	2.48	69	.016	.59
PC-PTSD-5^d^	3.07 (1.58)	2.82 (1.71)	3.30 (1.43)	1.27	69	.209	.30

^a^PSS4 = Perceived Stress Scale 4-item version.

^b^GAD7 = Generalized Anxiety Disorder Scale 7.

^c^PHQ8 = Patient Health Questionnaire 8.

^d^PC-PTSD-5= Primary Care PTSD Screen for DSM-5.

Participants reported their miscarriage was managed by “expectant only” (53.5%) and “medical and/or surgical” management (46.5%). A significantly lower percentage of the expectant management group (34.2%) was categorized in the good patient experience group compared to the medical/surgical management group (63.6%), *Χ*^2^ = 6.13, *p* = .013, Phi = .29 (small effect size). As shown in Table 4, within the medical/surgical management group, participants who had a good patient experience had significantly lower stress, anxiety, and depressive symptom scores than those with a poor patient experience and effect sizes were large. Within the expectant management group, there was no significant difference in psychological distress scores between participants who had good versus poor patient experiences, and effect sizes were small.

**Table 4:** Psychological Distress by Patient Experience Within Miscarriage Management Groups.

Scale	Medical/surgical management		*t*	*df*	*p*	*d*
	Good patient experience (*n* = 21)	Poor patient experience (*n* = 12)				
	*M (SD)*	*M (SD)*				
PSS4^a^	7.81 (2.79)	10.42 (3.80)	2.26	31	.031	.82
GAD7^b^	7.57 (5.80)	14.08 (4.64)	3.32	31	.002	1.20
PHQ8^c^	6.43 (6.06)	13.25 (8.23)	2.73	31	.010	.99
PC-PTSD-5^d^	2.90 (1.76)	3.75 (3.21)	1.51	31	.141	.55
Scale	Expectant management		*t*	*df*	*p*	*d*
	Good patient experience (*n* = 13)	Poor patient experience (*n* = 25)				
	*M (SD)*	*M (SD)*				
PSS4^a^	8.62 (1.89)	9.52 (4.06)	0.76	36	.454	.26
GAD7^b^	7.15 (3.87)	9.64 (6.13)	1.33	36	.193	.45
PHQ8^c^	7.15 (4.86)	9.40 (7.08)	1.02	36	.314	.35
PC-PTSD-5^d^	2.69 (1.70)	3.08 (1.55)	0.71	36	.484	.24

^a^PSS4 = Perceived Stress Scale 4-item version.

^b^GAD7 = Generalized Anxiety Disorder Scale 7.

^c^PHQ8 = Patient Health Questionnaire 8.

^d^PC-PTSD-5 = Primary Care PTSD Screen for DSM-5.

### Qualitative findings

Table 5 presents sociodemographic characteristics of good (n = 12) and poor (n = 6) patient experience groups and the total sample (N = 18) of participants who were interviewed. Most participants in both patient experience groups were White, had private insurance, an annual household income of ≥ $51,000, and lived in rural or suburban areas. All participants were married or partnered at the time of the study. Analysis of 18 transcripts led to the identification of four main categories, including *Access to Care*, *Healthcare Provider Interactions*, *Healthcare Support Staff Interactions*, and *Healthcare System Interactions*.

**Table 5:** Sociodemographic Characteristics of Interview Participants.

	Good (*n* = 12) n(%)	Poor (*n* = 6) n(%)	Total (*N* = 18) n(%)
Race			
Black or African American	0(0.0)	2(33.3)	2(11.1)
White	10(83.4)	4(66.7)	14(77.8)
Other	2(16.7)	0(0.0)	2(11.1)
Ethnicity			
Hispanic or Latina	2(16.7)	1(16.7)	3(16.7)
Not Hispanic or Latina	10(83.3)	5(83.3)	15(83.3)
Marital status			
Married/partnered	12(100.0)	2(33.3)	18(100.0)
Divorced/sep/widowed	0(0.0)	0(0.0)	0(0)
Highest level of education			
Less bacc	3(25.0)	2(33.3)	5(27.8)
Bacc or higher	9(75.0)	4(66.7)	13(72.2)
Annual Household Income			
≤ $50,000	2(16.7)	2(33.3)	4(22.2)
≥ $51,000	10(83.3)	4(66.7)	14(77.8)
Employment			
Full time	8(66.7)	4(66.7)	12(66.7)
Part-time/unemployed	4(33.3)	2(33.3)	6(33.3)
Insurance			
Private	10(83.3)	6(100.0)	16(88.9)
Medicaid/uninsured	2(16.7)	0(0.0)	2(11.1)
Residential Setting			
Rural	4(33.3)	1(16.7)	5(27.8)
Urban	0(0.0)	1(16.7)	1(5.6)
Suburban	8(66.7)	4(66.3)	12(66.7)

Age for the total sample ranged from 24–43 (*M* = 33, *SD* = 5.24). Age for good patient experience group (*M* = 32.92, *SD* = 5.85, *n* = 12) and poor patient experience group (*M* = 33.17, *SD* = 4.21, *n* = 6).

#### Access to care.

This category reflects participants’ ability to obtain obstetrics and gynecology (OBGYN) and mental health services, including geographical and logistical barriers, service availability, and the timing of care in the context of rural and suburban living and care availability during the COVID-19 pandemic.

Positive access experiences were described by participants who reported few to no barriers to obtaining care. Several participants reported that their provider prioritized timely visits and follow-up, in some cases due to their history of recurrent loss. One participant stated how being offered a virtual visit helped mitigate the burden of traveling long distances for prenatal care, “…which was really awesome and very comforting because [the clinic] was a 2-hour drive. So, I was thankful I didn’t have to make that drive, and I was just able to do it on the phone.”

In contrast, negative access experiences were described by participants who faced barriers related to provider shortages and limited service availability, as stated by one participant,

It’s typical up here on the mountain because there’s not enough doctors for the number of patients they have, there’s literally one clinic to serve three counties, so that’s not atypical, like you can’t even reach them on the phone, sometimes you have to go down there to make your appointment*.*

Women stated that challenges also included poor online reviews of local providers, the presence of a local emergency department without maternal services, and the absence of nearby specialists such as reproductive endocrinologists. They expressed that these issues forced them to drive anywhere between 30 minutes and 2 hours for quality OBGYN care.

The COVID-19 pandemic further intensified these access challenges. Even participants who had not previously had trouble accessing care reported delays in services during the pandemic. Participants described delays in being seen by providers as common, particularly due to quarantine requirements following COVID-19 exposure. Same-day appointments were mostly unavailable, and usual providers were often fully booked days in advance. A few participants also noted that OBGYN clinics reduced patient volume because of limited provider availability and concerns about COVID-19 transmission. Some clinics only saw patients with emergency cases and did not consider miscarriages to fall under that scope, as one participant explained, “It was very frustrating because I wasn’t able to get medical care because the doctor’s offices didn’t want to bring people in…The nurse said, ‘Just stay home, and if you have excessive bleeding, [then] go to the ER’.” Moreover, a couple of participants stated that mental health care was impacted by increased demand and referrals, and slow adoption of telehealth. One participant reported that although she had no difficulty accessing her OB-GYN, she was unable to connect with any mental health support, as virtual support groups and individual therapy services were unavailable. Furthermore, one participant who did manage to obtain therapy over the phone said that her sessions were interrupted by issues with phone service due to the rural location. Participants described how these issues combined led them to feel anxious, frustrated, dismissed, and alone.

#### Healthcare provider interactions.

This category reflects participants’ experiences engaging with healthcare providers, nurses, and medical and nursing students during miscarriage care in the context of the COVID-19 pandemic. This category encompassed how participants perceived communication, interpersonal engagement, clinical processes, and support across different moments of care, including diagnosis, decision-making, and follow-up. There are four sub-categories: *Provider and Nursing Interpersonal Behaviors*, *Effects of the COVID-19 Pandemic*, *Delivery of News*, and *Management Decision-Making*.

##### Provider and nursing interpersonal behaviors.

This sub-category captures descriptions of how healthcare providers, nurses, and trainees interacted with participants across care encounters. Table 6 presents a *focused* cross-case comparison of supportive and unsupportive provider and nursing interpersonal behaviors. When participants explicitly referenced nurses or nursing roles, these were separated; however, in cases where provider type was unclear, behaviors were categorized more broadly under “provider” to reflect the interaction rather than the specific role.

**Table 6:** Provider and Nursing Interpersonal Behaviors During Pandemic Miscarriage Care.

Provider Interpersonal Behaviors	
**Supportive**	**Unsupportive**
• Were kind, reassuring, and compassionate, treating participants as human beings (e.g., held hand, hug)	• Participants felt dismissed and rushed
• Presented all management options and let participants guide their care plan	• Made participants feel like another number, lacking empathy and compassion
• Removed any guilt or self-blame from participants	• Participants had to see unfamiliar providers
• Took time to answer all questions and provided time to process the news without rushing	• Failed to meet participants where they were mentally to receive information and make decisions
• Explained and provided guidance on physical symptoms during and after the loss, ensuring participants understood	• Did not educate participants on prevalence and causes of miscarriage, future fertility outlook, and secondary infertility risks
• Provided follow-up care	• No follow-up care provided
• Shared personal pregnancy loss experiences and sympathized	• Provided a packet of information and sent participants on their way
• Provided guidance on future fertility, pregnancies, medications, and menstrual cycles	• Inadequate explanation of management options and what happens to the baby’s remains after a D&C
• Offered additional confirmatory ultrasound and/or blood tests	• Did not inquire about participants’ mental or emotional state
• Offered notes for time off work for participants and their partners	• No formal at-home miscarriage collection kit provided with instructions
• Normalized the loss and explained the prevalence of miscarriages	• Did not educate on community resources such as support groups or coping strategies
• Acknowledged and validated participants’ feelings	• Failed to inform all care team members of participants’ diagnosis
• Including participants’ partners in the conversation over the phone or in person	• Partners were not included in the conversation via Facetime or phone
• Made participants feel heard and taken seriously	• Care was very clinical and by the book
• Built rapport and trust with participant	• Used insensitive terminology (e.g., “abortion,” “chromosomal misfire,” “you are young,” “can try again”)
• Provided support group information and other resources	• Did not provide thorough education to participants with recurrent miscarriages, assuming prior knowledge
	• Lost participant’s trust
**Nursing Interpersonal Behaviors**	
• Were kind, gentle, relaxed, and reassuring	• Seemed busy, interactions were brief and task-focused
• Provided anticipatory guidance on D&C procedure	• Took a long time to update participants, provide guidance, or answer questions
• Checked on participants via patient portal, answered questions, and offered support	• Made insensitive comments about having live children or that they could try again
• Treated participants with care, offering comfort and emotional support (e.g., patting, giving food, wiping tears)	• Were detached, cold, and barely present, ignoring participants’ pain and distress; bruised arm during IV insertion
• Shared personal pregnancy loss experiences and sympathized	• Asked participant for a urine sample for pregnancy testing
• Acknowledged participants’ reasons for being at the clinic to avoid repetition and re-traumatization	
• Provided frequent visits and updates on results	

Participants described positive encounters as those where providers and nurses were kind, compassionate, and took time to offer reassurance, answer questions, and provide thorough follow-up care, including extra confirmatory ultrasounds and/or blood tests. For example,

She was awesome, after the ultrasound, she came into the ultrasound room and she talked to us for a good 15 or 20 minutes about management, and how it wasn’t my fault and offered support, different things we could do as far as who we could talk to, provided us with resources, and then took us back to a patient room and discussed, even more, ‘If you need time off work please let me know, I’m giving you this note if you need more time, don’t hesitate to let us know’, even talked with my husband, just encouraged him as well.

Although references to medical and nursing students were infrequent, one participant described feeling respected when a medical student showed sincerity and respect.

There is one in particular, …he seemed like he cared, he walked in, and I was crying, and I can tell maybe it made him feel uncomfortable or he felt bad …but he gave me a moment, and he asked my permission if he could go into the OR to see the D&C and I thought that showed that he respected me.

In contrast, negative encounters often involved participants saying they felt like just a number or where providers and nurses were perceived as cold, detached, and lacking empathy, particularly when follow-up care and emotional support were absent. One participant shared,

He told me, I was having a spontaneous abortion, that I could try again like it wasn’t a big deal, he also told me I was young … I can have more kids and that really bothered me at the time, and they told me to go to my regular OBGYN in a few days to make sure my numbers had gone down and sent me out with a packet, and that was pretty much it.

Additionally, another participant shared that she felt uncomfortable when a nursing student stood silently at her doorway without engaging.

##### Effects of COVID-19 pandemic.

This subcategory reflects how healthcare provider interactions may have been shaped by the COVID-19 pandemic. Despite disruptions to care, some participants described providers who maintained high-quality, compassionate care during the pandemic. One participant described the experience as a “well-oiled machine” where clinical processes and protocols were maintained. Another participant shared that her provider granted special permission for both her and her partner to return together to view the imaging of a repeat ultrasound despite social distancing policies.

In contrast, several participants expressed that the COVID-19 pandemic introduced barriers to the care providers were able to offer. As one participant shared,

Honestly, the [biggest] thing I can say about the pandemic was just a complete lack of personalization, when I went in, it was immediate, he [partner] had to leave, and then, yeah, you’re in mask, and at the time, doctors were in full gowns, which is understandable, but it really felt so detached, it didn’t feel like I was with other people.

In one instance, a participant described how her care plan for an anticipated miscarriage was abandoned because providers were reluctant to see patients. Further, participants expressed feeling that miscarriages were overshadowed by COVID-19 concerns. One participant shared how she switched providers due to the provider’s anxiety and lack of knowledge about COVID-19 in pregnant patients, which increased the participant’s stress. In addition, two participants acknowledged that the overburdened healthcare providers could not offer the emotional support they needed during the pandemic miscarriage.

Yeah, they could have provided me a little bit more mental health and help,... as far as like the physical, they did everything they could. They were so stretched thin, I could see it on their faces already, and that was the beginning [of the COVID-19 pandemic]. They’re still battling that…they were so tired already, so I wish for things that they could not have possibly provided.

##### Delivery of news.

This sub-category focuses on how participants experienced provider interactions when receiving their diagnosis regarding their miscarriage. Most explained they had ultrasounds and/or blood work to confirm the diagnosis.

Several participants described receiving the news in a caring, compassionate, and empathetic manner. Although the delivery of the news was often described as matter-of-fact or “by-the-book,” some participants found this approach helpful, particularly when providers clearly explained what to expect and collaborated with them to develop a care plan. Others described providers who allowed time and space following the delivery of the news. One participant shared that “they told me I think in the nicest way they could” and noted that the provider offered her to stay in the room for as long as she needed. One participant shared,

She was just kind of like, ‘You know, it’s a, it’s another miscarriage. We’ll document it and be on the lookout for these things. You may feel this way. Call me back if this, this, and this’. And then we had, you know, the discussion about the anxiety and seeing my PCP… My OB is wonderful. She was very empathetic. And she kind of knows, through my first miscarriage, she remembers that one pretty well.

Conversely, several participants described aspects of the diagnostic process that heightened distress. Some providers waited for lab confirmation before giving the diagnosis, and participants shared how this waiting period was stressful. Some participants described simply receiving the news over the phone. Many shared how they were distressed about receiving the news alone due to partner restrictions, even if providers were compassionate in their delivery. One participant stated the following: “My doctor was wonderful and compassionate and could not have delivered this news better, but to not have my partner there in that moment was really hard. Others recounted receiving the diagnosis with limited explanation or emotional acknowledgment, as one participant shared,

I sat in the emergency room for hours upon hours for them to come in and tell me there is no heartbeat, your baby hasn’t grown since your last ultrasound. So of course, that started like a spiral. I called my OB the next morning, told them what had transpired, and at that point, the healthcare that I received diminished very, very quickly. My appointment with the OB was cancelled, and I was told to go see just a family practitioner that day. So went in and he said, ‘Yep, I’ve looked at your medical records from the ER, you definitely have a miscarriage, good luck,’ pretty much.

Another participant stated,

She was just like, ‘Well, I don’t know how to tell you this, but there’s no heartbeat.’ Like, there was no compassion. There was no trying to sugarcoat what she was about to say. It was just like, honestly, like a projectile vomit… I mean, I lost it, it was like an instant crying and devastation.

##### Management decision-making.

This sub-category captures participants’ experiences with how miscarriage care options were presented, discussed, and implemented following diagnosis, including how participants’ preferences and needs influenced management decisions. It reflects perceptions of involvement in decision-making, clarity of explanations, responsiveness to patient preferences, and satisfaction or dissatisfaction with how care plans were carried out.

Positive management decision-making experiences were described when participants felt informed, supported, and involved in choosing their care. Some participants described selecting dilation and curettage (D&C) as their first choice due to fears of miscarrying at home, a desire for a quicker or less traumatic process, or the ability to mark a specific anniversary of their baby’s passing. Others described making decisions based on personal circumstances, such as financial considerations. One participant shared,

So yeah, we were trying to save on finances, and I hate to even bring money into it, but I knew how much the surgery center bill would cost us, even with insurance.... So that’s one of the reasons why we decided on [Cytotec and Methergine].

While another participant said,

I took the medicine, and it failed, and that’s where I waited and waited and then had the D&C, which cost me $4000, so these are really expensive doctor’s visits too. [Then], in August 2020, I got lucky, and I had two rounds of the medication, and it worked.

Many participants also described positive experiences when providers offered clear recommendations tailored to their miscarriage history and individual physical and emotional needs. As one participant explained,

My doctor recommended [the D&C] because the miscarriage before that, …I had so much blood loss and so much pain, it was like labor pain, …I was starting to feel kind of dizzy and stuff. And she said, because of that, and with it being there’s more tissue [this time] and everything, that she didn’t want to make me go through that.

Some participants shared that they made their decision after providers thoroughly explained all options, answered questions, and scheduled follow-up appointments. For example,

I received absolutely 100% informed consent, I was given the options, and I was told the pros and cons of each and was not swayed either way. I was able to ask the questions that I had and was given the answer.

Alternatively, negative management decision-making experiences were described by several participants who mentioned being sent home to wait and see how their bodies would respond without much further support or being advised over the phone to stay home and visit the ED if bleeding became excessive. These participants felt they were left to manage the miscarriage on their own, with minimal follow-up or emotional support, adding to their distress.

#### Healthcare support staff interactions.

This category reflects participants’ experiences with healthcare support staff during miscarriage care, including interactions with chaplains, environmental service staff, ultrasound technicians, pregnancy center staff, volunteers, and receptionists. Participants described a range of both supportive and unsupportive interactions that influenced their overall care experience.

Positive staff interactions were described when participants felt emotionally supported, acknowledged, and cared for during vulnerable moments. One participant said that she received an unexpected visit from a chaplain who happened to be a familiar face. The chaplain sat with her, rubbed her hand, and took time to slow things down for her so she could process it. Some participants described how their interactions with healthcare support staff were more meaningful than any other interactions they had, as seen when one participant shared her interaction with a phlebotomist: “she was super nice, and very, she helped me feel more relaxed than anybody else probably did”. Similarly, another participant shared that the most compassion she received during her hospital visit came from an environmental service staff member:

The most compassion I actually got from somebody at that hospital that day was a custodian who walked by my door … with me bawling, and she looked at me, ‘Are you OK?’. And I just kept bawling, she came in that room and hugged me.

Additionally, a couple of participants shared their positive experiences with pregnancy crisis center staff who provided extensive support, including information, emotional support, support group invitations, reassurance, prayer, and ultrasounds at no cost. One participant shared the following: “I feel that it would have helped me a lot in that moment when I lost my baby, they gave me a lot of information. They invited me to go to the group...”. This participant also stated that going to the pregnancy center alleviated her anxiety, especially given her unemployment and financial constraints. Another participant shared that because many women at the pregnancy crisis center had similar personal experiences, it helped reduce feelings of isolation: “They wanna be there to be a mentor for other women that have experienced miscarriages or that have had abortions or in a bad situation, so I just, I know I’m not alone anymore.”

Participants also described varied interactions with ultrasound technicians. In some cases, participants shared that the ultrasound technician delivered the news of the loss, especially when participants had prior miscarriages and knew what to look for, were friends with the technician, or suspected a miscarriage based on symptoms. Some found these encounters supportive; for example, one participant shared that she valued the technician’s professionalism and sensitivity, especially given her history of miscarriages. Another participant appreciated the technician’s condolences and hug, sharing: “They never made me feel rushed. Even though it was like during the pandemic, she still gave me a hug. So, I felt like they were grieving with me”.

On the other hand, negative interactions with the ultrasound technician were described by some participants who felt uncomfortable, humiliated, or neglected. One participant found the interaction very matter-of-fact, while another participant shared that she suffered humiliation from an incorrect ultrasound probe insertion. Another participant felt she had been left alone and undressed for what “seemed like an eternity” without receiving any information. Negative interactions with other support staff were also reported. One participant described her encounter with a receptionist, explaining,

She didn’t know what I was going through, and it’s not her fault that I was exposed to [COVID-19] or the fact that I was having a miscarriage, but just the fact that she was so… it was like I didn’t belong there, and I didn’t need to be there at that moment. You could tell she was kind of, I don’t want to say standoffish, but very like I had the plague kind of thing.

#### Healthcare system interactions.

This category reflects participants’ experiences with organizational processes and policies encountered while seeking or receiving miscarriage care, such as hospital procedures and policies, COVID-19 screening and visitor policies, documentation practices, insurance and billing processes, and emergency department (ED) care. Participants described both positive and negative system-level experiences that shaped their overall care.

Positive system-level experiences were described by a few participants when hospital procedures and communication processes helped them feel informed, supported, and spared from additional emotional burden, even in the context of COVID-19 restrictions. One participant shared that she was comforted knowing that her spouse would be able to receive real-time updates regarding her procedure at the hospital.

They made him wait in the waiting room, they had this huge TV board that had a portion of the person’s first and last name, so it could show my husband throughout this procedure what the steps I was going through. So that was comforting for me that he knew what was going on.

One participant noted that she was admitted to a non–mother-baby unit, which she perceived as a thoughtful decision to reduce emotional distress from hearing live babies. Another participant described feeling reassured when providers told her that they were already informed about the reason for her visit, saying, “so that made me feel a little bit better that I didn’t have to say, like ‘I’m here because I think I’m miscarrying or I’m scared’, I didn’t have to say it out loud again”.

Negative system-level experiences were frequently linked to COVID-19–related screenings procedures as well as policies that restricted partner presence during clinic visits, diagnostic appointments, and procedures. A few participants described system-level screening processes during COVID-19 as both understandable and distressing. One participant acknowledged the purpose of repeated COVID screening questions but described feeling frustrated having to follow these procedures while she was more concerned about losing her pregnancy. Most participants described heightened distress when receiving their diagnosis or undergoing treatment without their partner’s support due to COVID-19-related visitor restriction polices. As one participant explained, “So of course I had to find out [about the miscarriage] in the emergency room alone because of covid protocol, and then all of the follow-up appointments, I had to go to alone, which was very difficult.”

Additional negative experiences were associated with administrative, documentation, and billing processes. Two participants mentioned that the term “abortion” in their medical and discharge papers was hurtful because it is often associated with elective procedures and did not accurately represent their situation. Due to this terminology, one of these participants said that her insurance billed her for an elective procedure, leading to an additional struggle with her human resources director to resolve the issue. Another participant reported that a previously established care plan was abandoned, resulting in her miscarriage not being documented in the medical record and creating barriers to future care and insurance coverage. Furthermore, a couple of participants described distress related to the timing of insurance billing, as one participant shared,

Like ‘Oh my God, I just had two miscarriages back-to-back’, and now they’re sending me this ginormous bill, it took them like a week, and I’m like, ‘Who does that? Can I please at least grieve the loss of my child?’

Lastly, a couple of participants shared that distress was also caused by long wait times at the ED. One participant described the experience as terrible, being left for hours in the exam room before an ultrasound was performed. She said she felt overwhelmed by the chaotic environment and the uncertainty of not knowing her pregnancy status or what to expect. Another participant said she was only admitted sooner because she began actively bleeding in the lobby,

So, I didn’t get seen immediately, but once I started gushing out more blood and bleeding through the wheelchair and my other pair of clothes, that’s when it’s like, ‘OK, we gotta get her back’. And once I got back, it was like waiting for hours and hours…with the nurses back there, they took forever, I didn’t really get questions answered, and I’m like, ‘Oh my God’, I really just want to be with my husband and I felt like the wait for hours was uncalled for, being that it was such an urgent matter and there was like a death happening.

### Data integration

We integrated qualitative and quantitative findings for the interviewed subsample (n = 18) using a joint display to examine levels of convergence between qualitative categories and subcategories, and quantitative patient experience classifications. Table 7 presents the distribution of positive and negative qualitative evaluative direction within each category and sub-category across participants classified as having good or poor quantitative patient experiences. “No Report” indicates categories or subcategories that were not discussed during interviews. Degree of convergence is depicted in the final column.

**Table 7:** Convergence of Qualitative Categories With Quantitative Patient Experience Classification.

	Quantitative Patient Experience Classification						
	Good Experience *n* = 12		Poor Experience *n* = 6		No Report^a^ *N* = 18		Convergence Level^b^
**Qualitative Category**	n	%	n	%	n	%	
**Access to Care**					0	0	67% Moderate
Positive^c^	8	67	2	33			
Negative^c^	4	33	4	67			
**Healthcare Provider Interactions**							
Provider and Nursing Interpersonal Behaviors					0	0	89% Strong
Positive^c^	10	83	0	0			
Negative^c^	2	17	6	100			
Effects of COVID-19 Pandemic					9	50	89% Strong
Positive^c^	3	75	0	0			
Negative^c^	1	25	5	100			
Delivery of News					0	0	78% Strong
Positive^c^	9	75	1	17			
Negative^c^	3	25	5	83			
Management Decision-Making					0	0	78% Strong
Positive^c^	11	92	3	50			
Negative^c^	1	8	3	50			
**Support Staff Interactions**					9	50	56% Weak
Positive^c^	3	60	2	50			
Negative^c^	2	40	2	50			
**Healthcare System Interactions**					1	6	53% Weak
Positive^c^	4	36	1	17			
Negative^c^	7	64	5	83			

^a^‘No Report’ indicates that the category or sub-category was not discussed by participants during interviews; these cases were excluded from percentage and convergence calculations.

^b^Convergence level is the percentage of reporting participants with aligned findings: positive accounts within the good experience group or negative accounts within the poor experience group. Levels were classified as weak (50%–59%), moderate (60%–74%), or strong (≥75%).

^c^Positive and negative designations represent evaluative direction used for convergence assessment, not additional qualitative subcategories.

The four subcategories of the healthcare provider interactions category demonstrated strong convergence (≥75% alignment) between qualitative narratives and quantitative classification. Participants quantitatively classified as having a good patient experience most frequently described positive qualitative accounts for provider and nursing interpersonal behaviors, effects of COVID-19, delivery of news, and management decision-making. In contrast, those classified as having a poor experience most frequently described negative qualitative accounts within provider and nursing interpersonal behaviors, effects of COVID-19, and delivery of news. For the management decision-making subcategory, qualitative responses among the poor experience group were evenly divided.

The access to care category demonstrated moderate convergence, indicating meaningful but less consistent alignment between qualitative and quantitative findings. Participants in the good experience group more often described positive accounts. In contrast, those in the poor experience group more frequently reported negative accounts.

The categories of healthcare support staff interactions and healthcare system interactions demonstrated weak convergence. For healthcare support staff interactions, participants in the good experience group more often described positive experiences, whereas responses in the poor experience group were evenly split (50% positive, 50% negative). Healthcare system interactions were more frequently described negatively across both groups, particularly among those classified as having a poor experience. High “no report” rates were observed for effects of COVID-19 and support staff interactions, as these domains were not consistently discussed across interviews.

## Discussion

In this mixed-methods study, we examined the patient experiences of women who miscarried a desired pregnancy during the COVID-19 stay-at-home mandates in North Carolina and explored relationships of patient experience with miscarriage management, psychological distress, and COVID-19–related stressors. Quantitative findings identified distinct patient experience groups and demonstrated differences in psychological distress and miscarriage management across these groups. Qualitative interviews provided contextual insight into women’s experiences of miscarriage care during this period, including access to care and interactions with healthcare providers, support staff, and the healthcare system.

Participants were nearly evenly distributed between good and poor patient experience classifications. Sociodemographic differences suggest that women classified in the poor experience group were more likely to report significantly lower household income and less stable employment compared to those in the good experience group. Possible explanations include limited paid leave, inflexible work schedules [21], transportation or childcare constraints, fewer financial resources to access care, and provider implicit bias [37]. Although the study was not designed or powered to examine social or structural determinants of patient experience, these patterns warrant further investigation, particularly given known disparities in reproductive healthcare access and outcomes [37]. Access-related stressors were infrequently endorsed in the survey and did not significantly differentiate patient experience groups. In interviews, several women described minimal barriers to obtaining obstetric care. However, others reported delays, reduced appointment availability, telehealth disruptions, and challenges accessing mental health services during the pandemic. Notably, these access challenges were described across both good and poor patient experience groups. This pattern suggests that participants may have distinguished between system-level access constraints and the quality of interpersonal care when evaluating their experience. Additionally, normalization of limited access among participants residing in rural or suburban areas may have influenced how these barriers were interpreted. Prior research has documented persistent disparities in obstetrical and reproductive healthcare access in rural populations [38], and evidence suggests that the COVID-19 pandemic may have compounded existing access challenges [20].

Significant differences emerged across nearly all patient experience survey items, validating the cluster-derived group distinctions. Participants in the good experience group endorsed higher rates of clear explanations, feeling listened to, respect, adequate time, emotional support, follow-up scheduling, and information provision. Notably, across the total sample, the lowest endorsements were for “provider addressed emotional needs” and “provided information on support groups, therapy, or counseling services”, indicating gaps in psychosocial support regardless of overall patient experience classification. Consistent with prior research, empathetic communication, active listening, and follow-up have been identified as critical components of supportive miscarriage care, whereas insensitive language, limited continuity, and inadequate follow-up contribute to distress [6,20].

Importantly, quantitative findings demonstrated that poorer patient experiences were associated with screening scores meeting established thresholds for clinically significant anxiety and depressive symptoms, as well as higher perceived stress. These findings suggest that patient experience may extend beyond perceptions of care and relate to psychological well-being following miscarriage, consistent with prior research demonstrating associations between communication quality and mental health outcomes [39]. In contrast, PTSD scores did not differ significantly between patient experience groups. Notably, nearly two-thirds of the total sample screened positive for likely PTSD, indicating a high overall burden of trauma-related symptoms following miscarriage. This elevated prevalence across both groups may have limited variability between classifications and reduced the ability to detect differences by patient experience.

Further, while students’ presence is beneficial for educational purposes, it is important to balance this with patients’ emotional needs. Study findings emphasize the ethical importance of obtaining patient consent, particularly during sensitive procedures like miscarriage management. One study found that OB/GYN patients reported lower rates of these practices compared to patients in general surgery or infectious diseases and were most uncomfortable with the presence of medical students, indicating a need for improvements in respecting patients’ fundamental rights [40].

The COVID-19 pandemic formed an important contextual backdrop for participants’ miscarriage experiences. In qualitative interviews, a few participants described providers as being stretched thin and navigating heightened demands, consistent with other reports [41]. Some participants perceived that pandemic-related pressures limited providers’ capacity to offer emotional support. Quantitative findings demonstrated that deficits in interpersonal behaviors were associated with poorer patient experiences and higher psychological distress; however, this study did not directly examine whether pandemic-related strain contributed to these associations. Taken together, these findings suggest that system-wide pressures during the pandemic may have influenced how miscarriage care was experienced. These observations highlight the importance of ensuring adequate provider support during periods of healthcare strain.

One potential approach that has been proposed in the literature is the implementation of trauma-informed care (TIC). By recognizing and responding to signs of trauma in patients and providers, healthcare systems may foster environments that support emotional safety and resilience [42]. When adopted at a system level, TIC has been described as a framework that may help preserve compassionate care during periods of heightened demand [43].

Miscarriage management type was associated with differences in patient experience, with individuals undergoing expectant management less likely to report a good patient experience than those receiving medical or surgical management. Within the medical and/or surgical management group, participants who reported a good patient experience also reported lower levels of stress, anxiety, and depressive symptoms compared to those reporting a poor experience. In contrast, among participants undergoing expectant management, psychological distress did not differ by patient experience classification. These findings suggest that the relationship between patient experience and psychological distress may vary by miscarriage management type.

Qualitative narratives provide possible context, as participants undergoing expectant management described prolonged uncertainty, limited follow-up, and being sent home with minimal guidance, which may shape how distress is experienced regardless of patient experience classification. Prior randomized controlled trials have reported no overall differences in psychosocial outcomes by miscarriage management type [9,10]. Our findings build on this literature by suggesting that, while management type alone may not determine psychological outcomes, the association between patient experience and psychological distress may operate differently across management types. It is also possible that completion of medical or surgical management shortens the period of uncertainty and physical symptoms, thereby reducing distress independent of patient experience with their provider [44].

Qualitative interviews revealed that support staff encounters were meaningful for some participants. Women described positive interactions characterized by comfort through gestures such as hugs, prayer, and handholding, while negative experiences included unwelcoming attitudes, procedural errors, and delayed communication. Although support staff interactions were not consistently discussed across interviews, these encounters were salient for some participants. Prior research similarly recognizes the importance of training all members of the healthcare team, including support staff, on empathy and effective communication [6,15].

Healthcare system-level processes and policies also emerged as contextual factors shaping miscarriage experiences. Participants described distress related to COVID-19–related visitor restrictions, administrative procedures, documentation terminology, billing timing, and emergency department wait times. These processes represent modifiable factors that may influence miscarriage patient experiences, even when care is perceived positively. Most survey participants identified the inability to have a support person at healthcare visits as a stressor, which may explain the lack of significant differences between patient experience groups. Our qualitative findings reinforced this, as many women stated feeling unsupported and distressed due to having to attend their healthcare visits alone. Similar patterns have been documented elsewhere; for example, 92% of women in a United Kingdom study reported attending antenatal appointments alone during the COVID-19 pandemic, contributing to significant distress [45]. Support persons, especially partners, are valued by women experiencing a perinatal loss [20,46] and by pregnant women during routine visits [47]. Healthcare system interventions, such as alternative communication strategies including telehealth visits, phone calls, or real-time updates (e.g., lobby displays or text messaging), may help mitigate patient and family experience and reduce anxiety when physical presence of support persons is not feasible, particularly during periods of restricted visitation [48].

Participants also described distress and confusion related to discharge documentation using the term “spontaneous abortion” and early billing communications following miscarriage, indicating opportunities to delay non-urgent billing processes and adopt more patient-centered language in patient-facing discharge documentation and Electronic Health Record (EHR) communications. Prior research demonstrates that terminology used to describe non-viable pregnancy is shaped by stigma and varies in clarity and acceptability, with patients preferring terms such as “miscarriage” or “early pregnancy loss” and rating “spontaneous abortion” as the least clear and least preferred [49,50]. Additionally, placement in mother-baby settings designed for postpartum patients has been shown to intensify emotional distress after pregnancy loss, supporting recommendations for more sensitive unit assignment [51]. Emergency department wait times were described by several participants as particularly distressing. Women recounted waiting for extended periods while experiencing active bleeding, uncertainty about pregnancy status, or confirmation of the loss. These accounts suggest that timeliness and communication in emergency settings may meaningfully shape miscarriage experiences, particularly when patients present with acute symptoms [52].

The integration of qualitative and quantitative findings reflects the subsample of 18 women interviewed and should be interpreted in the context of the full quantitative results and broader qualitative themes discussed above. Strong convergence was observed in provider and nursing interpersonal behaviors, delivery of news, management decision-making, and effects of COVID-19 (among those who discussed this). Notably, this pattern is consistent with the patient experience survey items, which focused on communication, respect, emotional support, being listened to, time spent, and follow-up. Access to care demonstrated moderate convergence, with positive accounts more common in the good experience group and negative accounts more common in the poor experience group. However, the alignment was less consistent than in interpersonal domains, suggesting that while logistical and structural factors influenced patient experience, they may not have been as determinative of overall experience ratings as relational aspects of care.

In contrast, healthcare support staff interactions and healthcare system interactions demonstrated weak convergence. Support staff interactions were more often described positively among participants in the good experience group, whereas responses in the poor experience group were evenly divided. Healthcare system interactions were frequently described negatively across both groups, particularly among those classified as having a poor experience. These findings suggest that system-level frustrations may be widespread and not exclusively associated with overall patient experience classification. Additionally, high “no report” frequencies in effects of COVID-19 and healthcare support staff interactions suggest that these domains were not consistently raised across interviews and may have been less central to participants’ overall evaluations compared to provider-centered interactions. However, the interviews did not explicitly probe the effects of COVID-19 on healthcare providers or experiences involving healthcare system and support staff interactions. Therefore, lower reporting may reflect the structure of the interview guide rather than the lesser importance of these experiences.

### Limitations

Several limitations should be considered when interpreting these findings. Although recruitment occurred through multiple channels, the use of convenience and snowball sampling may have introduced self-selection bias, as women who were more willing to discuss their miscarriage experience may have been more likely to participate. Additionally, although we aimed to include women of diverse backgrounds, most participants identified as non-Hispanic White.

A primary limitation is that the cross-sectional design and 14–31 month recall period limit the interpretation of directionality or causality of the association between patient experience and psychological distress. Although poorer patient experiences were associated with higher levels of distress symptoms, it is also possible that higher psychological distress influenced how participants perceived and reported their patient experiences, suggesting that the relationship may be bidirectional. In addition, we did not collect data at the time of the miscarriage, which could have offered more immediate insights into their psychological distress and patient experience.

Although participants were asked specifically about care received from registered nurses, physicians, and advanced practice nurses, many were unable to recall or distinguish between provider roles. Additionally, interviews did not explicitly probe experiences related to COVID-19 effects on the healthcare provider, healthcare system interactions, or healthcare support staff interactions. Consequently, findings related to these categories and sub-categories reflect experiences that participants raised spontaneously and may not fully capture the breadth of these aspects of care.

### Policy and practice implications

Identifying pandemic-specific barriers to healthcare access is crucial for informing future strategies to enhance healthcare delivery and integrate mental health support within OBGYN services during global crises. Policy changes are needed, particularly in rural and suburban areas, to ensure equitable access to OB/GYN and mental health services through mental health hotlines, digital platforms, and improved phone and internet connectivity. Policies supporting partner presence during medical visits, even virtually, could alleviate some emotional distress in situations where physical presence may not be feasible. Standardizing accurate yet sensitive terminology in medical records and discharge documentation, along with delaying non-urgent billing communications, may help reduce avoidable emotional distress. Additionally, efforts to improve emergency department triage processes and communication during prolonged waits may enhance patient experiences in acute miscarriage presentations.

Establishing standardized miscarriage care protocols that include routine follow-up visits and psychological distress screenings using validated tools is essential to support women after miscarriage and address their mental health needs, particularly for those undergoing expectant management who may have less frequent contact with healthcare providers. Shared decision-making models that explicitly address emotional readiness, access to support, and patient preferences, rather than focusing solely on clinical appropriateness, may improve patient experiences and psychological outcomes following miscarriage. Implementing TIC practices may help recognize and address trauma in patients and providers, avoiding re-traumatization and improving patient experiences. Mental health resources and support for healthcare providers are essential to mitigate burnout and compassion fatigue, ensuring they can offer empathetic care. Enhanced training for all healthcare staff, including non-clinical workers, in empathy, effective communication, and sensitive terminology could significantly improve patient interactions, particularly during a time of heightened stress and grief.

### Future research

Future research should encompass a larger and more diverse population to identify specific barriers to care and support needs following miscarriage during global crises. Multivariable analyses with larger sample sizes are needed to clarify the role of income and employment status in shaping patient experience after pregnancy loss. Longitudinal studies would be valuable in examining how access to medical and mental health support impacts the long-term mental health outcomes of women who have experienced miscarriage(s) amid a global crisis. Additional research is also needed to assess the effectiveness of training programs for healthcare providers and support staff focused on empathy, communication, and the use of sensitive terminology, as well as the impact of implementing TIC on provider burnout and patient experience.

Future studies should also more explicitly examine the role of support staff and system-level processes, such as support staff interactions, documentation practices, and billing timing, in shaping patient experiences following miscarriage. Research evaluating structured follow-up interventions, particularly for individuals undergoing expectant management, is needed to determine whether scheduled check-ins and clearer guidance reduce prolonged uncertainty and psychological distress. Finally, findings from this study highlight the need for longitudinal studies beginning near the time of miscarriage to clarify the potentially bidirectional relationship between patient experience and psychological distress. Such studies could determine whether differences in psychological outcomes are related to management experiences, the timing and completion of the miscarriage process, pre-existing or concurrent distress, or a combination of these factors.

## Conclusion

This study aimed to understand the patient experience of women in NC following the miscarriage of a desired pregnancy during the COVID-19 pandemic. Provider and nursing interpersonal behaviors, including communication, emotional support, respect, time spent, and follow-up, were closely connected to overall patient experience and psychological distress. The relationship between patient experience and psychological distress may operate differently across miscarriage management types, particularly when care involves prolonged uncertainty or limited follow-up.

While pandemic-related disruptions and healthcare system constraints formed an important contextual backdrop, provider and nursing-centered interactions appeared particularly influential in how women interpreted their care. These findings emphasize the importance of interdisciplinary, empathetic, and trauma-responsive miscarriage care. Organizational policies that support sensitive terminology, thoughtful communication practices, timely follow-up, and attention to modifiable healthcare processes may improve patient experiences and reduce avoidable distress, particularly during periods of healthcare strain or restricted access.

## Supporting information

S1 File Bilingual semi-structured interview guide. (DOCX)
