# Supplementary material for: Navigating grief and healthcare: Mixed-methods study on patient experiences of miscarriage during COVID-19
Source: PLoS One. 2026 Sep 24;21(9):e0359158. doi: 10.1371/journal.pone.0359158 (PMC13606936; doi:10.1371/journal.pone.0359158)
Supplement: Supplementary file 1 [file pone.0359158.s001.docx]

# **S1 File. Bilingual semi-structured interview guide**

This interview guide was previously published within the Measures/Interview Supplementary File accompanying Fernandez-Pineda M, Swift A, Dolbier C, Guard Banasiewicz K. Compounding stress: A mixed-methods study on the psychological experience of miscarriage amid the COVID-19 pandemic. BMC Pregnancy Childbirth. 2024;24:426. https://doi.org/10.1186/s12884-024-06610-z. The interview guide is reproduced without changes to its content under the Creative Commons Attribution 4.0 International License (<https://creativecommons.org/licenses/by/4.0/>). The unrelated survey measure included in the original supplementary file has been omitted, and formatting has been adjusted for the current manuscript.

**In-depth Interview Guide**

***Guía de Entrevistas***

1. Tell me about your experience of having a miscarriage. *Cuénteme sobre su experiencia de tener un aborto espontáneo.*

2. How has the COVID-19 pandemic affected you and your experience?*¿Cómo le ha afectado la pandemia de COVID-19 a usted y a su experiencia?*

3. Did you feel alone or lonely during the loss?

*¿Se sintió solo o solitario durante la pérdida?*

4. Did you visit an ER or an OB ER at any time during that pregnancy or during/after the loss? What was that experience like for you?

*¿Visitó una sala de emergencias u obstetricia en algún momento durante ese embarazo o durante o después de la pérdida? ¿Cómo fue la experiencia para ti?*

5. How was the news of the loss delivered to you/ how did you come to know about the loss? How was your miscarriage managed and how did you feel about that? *¿Cómo le fue entregada la noticia de la pérdida? ¿Cómo se manejó su aborto espontáneo y cómo se sintió al respecto?*

6. Do you wish that something about your care would have been different?*¿Desea que algo de su cuidado hubiera sido diferente?*

7. How have you and your partner/support person coped with the loss? How do you feel about the miscarriage ***now***?

*¿Cómo han afrontado usted y su pareja / persona de apoyo la pérdida?¿Cómo se siente* ***ahora*** *con respecto al aborto espontáneo?*

8. Would you have been interested in participating in some type of therapy or intervention for coping and healing? If you were to participate in a type of therapy or intervention, how would that look like to you?

*¿Habría estado interesado en participar en algún tipo de terapia o intervención para afrontarlo y curarlo? Si tuviera que participar en un tipo de terapia o intervención, ¿cómo le parecería eso?*

9. Are you anxious or nervous about the future in regard to pregnancy? Would you try or do anything differently in the future? What would you prefer your care to look like moving forward?

*¿Está ansiosa o nerviosa por el futuro con respecto al embarazo? ¿Intentarías o harías algo diferente en el futuro? ¿Cómo preferiría que fuera su atención en el futuro?*

10. Miscarriage is often something that is not openly talked about. Is there something you wish people would ask you about or that you could share with others about your experience?

*El aborto espontáneo es a menudo algo de lo que no se habla abiertamente. ¿Hay algo sobre lo que desearía que la gente le preguntara o que pudiera compartir con otros sobre su experiencia?*
